# The Role of Cardiac MRI in Pulmonary Hypertension- Is it Still an Underutilized Tool

**DOI:** 10.2174/0118743064288565240515115239

**Published:** 2024-06-04

**Authors:** Prakash Banjade, Ashish Subedi, Sampada Acharya, Asmita Itani, Munish Sharma, Nadeem Kassam, Shekhar Ghamande, Salim Surani

**Affiliations:** 1 Department of Medicine, Manipal College of Medical Sciences, Pokhara, 33700, Nepal; 2 Department of Medicine, Gandaki Medical College, Pokhara, 33700, Nepal; 3 Department of Medicine, Chitwan Medical College, Bharatpur, 44200, Nepal; 4 Department of Medicine, Institute of Medicine, Tribhuvan University Teaching Hospital, Kathmandu, 44600, Nepal; 5 Division of Pulmonary, Critical Care and Sleep Medicine, Baylor Scott and White, Texas, USA; 6 Department of Medicine, Aga Khan University, Nairobi, 30270, Kenya; 7 Division of Pulmonary, Critical Care and Sleep Medicine, Adjunct Clinical Professor, Texas A & M University, Texas, USA

**Keywords:** Pulmonary hypertension, Right heart failure, Cardiac magnetic resonance imaging, Right ventricle failure, Echocardiogram in pulmonary hypertension, Right heart catheterization

## Abstract

Pulmonary hypertension (PH) is an intricate medical issue resulting from increased pressure in the pulmonary artery (PA). The current gold standard for diagnosis involves an invasive procedure known as right heart catheterization. Nevertheless, cardiac magnetic resonance imaging (cMRI) offers a non-invasive and valuable alternative for evaluating the function, structure, and blood flow through the pulmonary artery (PA) in both the left ventricle (LV) and right ventricle (RV). Additionally, cMRI can be a good tool for predicting mortality by assessing various hemodynamic parameters. We perceive that cMRI may be an underutilized tool in the evaluation of PH. More discussions might be needed to highlight its utility in patients with PH. This article aims to discuss the potential role of cMRI in evaluating PH based on the review of recent literature.

## 1 CORE TIPS

The diagnosis of PH can be challenging due to its initial non-specific signs and symptoms. Although there are many diagnostic methods available, they all have certain limitations. cMRI is a promising adjunctive tool that can provide diagnostic and prognostic information by analyzing functional, structural, and velocity data. This valuable and non-invasive method demonstrates high sensitivity to alterations in morphology, function, and flow-related factors while also exhibiting strong reproducibility. Despite these capabilities, cardiac magnetic resonance imaging (cMRI) has yet to be fully utilized in current clinical practice. We suggest conducting additional research to explore the potential of employing cMRI in the management of patients with pulmonary hypertension (PH).

## 2 INTRODUCTION

PH is a potentially serious condition that occurs due to an increase in pressure in the PA, causing progressive right ventricular dysfunction. It affects approximately 15 individuals per 1 million in the population. It is more prevalent in females, with a ratio of 1.5:1 to 4.1:1 [1]. Pulmonary hypertension (PH) is linked to considerable levels of morbidity and mortality. Irrespective of its origin, it adversely affects the quality of life [2]. Despite contemporary treatment approaches, it may still be correlated with a reduction in life expectancy. To diagnose PH, the standard test is right-sided heart catheterization (RHC). Patients with PH have a resting mean pulmonary arterial pressure (mPAP) of 20 mm Hg or greater [3]. To assess whether a patient has PH, guidelines recommend using echocardiography to determine if they are at low, medium, or high risk. The reason for this is that right heart catheterization (RHC) is an invasive test with potential complications [3]. Other imaging modalities include CT scans, ventilation-perfusion (V/Q) scintigraphy, and cMRI. All these techniques have their advantages and disadvantages [4].

cMRI provides a comprehensive assessment of both structure and function of the chambers of the heart and pulmonary vasculature. During the procedure, imaging can unveil morphological changes in the heart, such as an increased diameter of the pulmonary artery, artifacts in pulmonary flow, and thickening of vessel walls. These changes can be identified through the use of black-blood T1-weighted sequences [5]. Cine steady-state free-precession (SSFP) sequences can assess ventricle geometry and function, aiding in the early detection of right ventricle (RV) dysfunction and left ventricle (LV) involvement. They also furnish information about valvular function. Magnetic resonance imaging (MRI) markers like RV end-diastolic volume (RVEDV), RV systolic volume (RVSV), and interventricular septal bowing serve as valuable prognostic parameters [6-8]. Accurately predicting and monitoring the progression of pulmonary arterial hypertension (PAH) is crucial for providing optimal treatment to patients 9 [9]. Cardiac MRI has both diagnostic and prognostic value, making it an important imaging modality. In this review, we aim to visit the role of cMRI in PH based on recent literature available.

### 2.1 Pulmonary Hypertension: Causes and Classification

The World Health Organization (WHO) categorizes pulmonary hypertension (PH) into five distinct groups based on its cause. These groups are pulmonary artery hypertension (PAH), pulmonary hypertension due to left-sided heart disease, pulmonary hypertension due to lung disease and hypoxia, pulmonary hypertension due to pulmonary artery obstructions, and pulmonary hypertension with unknown and/or multiple causes [10, 11]. PH can also be classified as precapillary, post-capillary, and combined pre- and post-capillary. Precapillary PH, which is characterized by pulmonary artery wedge pressure (PAWP) ≤15 mmHg, is caused by the remodeling of pulmonary vessels that leads to increased resistance in pulmonary vessels. Post-capillary PH is associated with heightened pulmonary venous pressure in left-sided heart conditions, with PAWP>15mmHg [12].

### 2.2 Cardiac Changes in PH

Elevated pressure in the pulmonary artery puts a strain on the right ventricle (RV), hampers blood flow in the pulmonary veins, and diminishes the volume of blood reaching the systemic circulation. In response to the heightened pulmonary artery pressure, remodeling of the right ventricular wall initiates as a countermeasure.

**Fig. (1):**
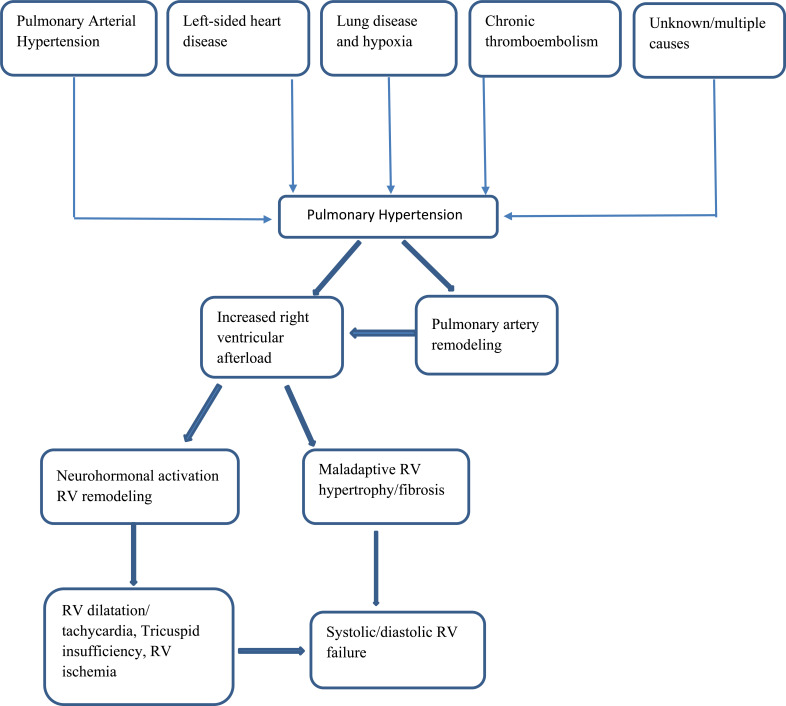
Summarizes the pathophysiology of pulmonary hypertension.

The muscles of the right ventricle are not accustomed to moving blood against the high pressure in the lungs, so they begin to weaken over time. Development involves a series of processes, such as extending and modifying RV geometry. As the disease progresses and afterload increases, the right ventricle undergoes adaptive remodeling to maintain the right cardiac output by increasing contractility. Depletion of compensatory right ventricular remodeling (RV-PA uncoupling) ultimately leads to mismatch and increased heart volume, leading to heart failure [13]. The interventricular septum flattens or reverses its curvature, a condition known as interventricular septal bowing. The enlargement of the right ventricle (RV) causes the tricuspid valve annulus to dilate, leading to functional tricuspid regurgitation. This adds an additional challenge of volume overload to an already high-pressure-loaded RV. Subsequently, there is an increase in pressure within the right atrium, dilation of the right atrium, a distended inferior vena cava, and the development of pericardial effusion in a sequential manner (Fig. **1**). In this advanced stage, the elevation of RV end-diastolic pressure serves as a survival marker in pulmonary hypertension (PH) [14].

### 2.3 Commonly Used Diagnostic Modalities and their Limitations

Due to the lack of specificity in the initial symptoms and signs, diagnosing PH in a patient may be difficult with a routine medical examination. A high clinical index of suspicion is needed to diagnose the disease before irreversible pathophysiologic abnormalities occur. Right heart catheterization is the current guideline recommended diagnostic tool of choice, which provides reliable measurement of pulmonary artery pressure. Echocardiography, CT scan, Ventilation-Perfusion (V/Q) scintigraphy, and cardiac MRI are frequently employed diagnostic tools. Table **1** outlines certain drawbacks associated with the existing imaging methods for pulmonary hypertension (PH).

While RHC is a dependable way to assess PAP, it falls short of providing a thorough evaluation of the right ventricle's condition. This limits critical data assessment, such as right ventricle-pulmonary artery coupling and left ventricle-right ventricle interdependence. Additionally, RHC is an invasive procedure that poses potential risks of serious complications, such as bleeding, pneumothorax, infection, pulmonary artery rupture, and heart block [15, 16]. While evaluating PH with RHC, patients may be dehydrated and have decreased intravascular volume, which could result in an incorrectly low PWP [16].

Transthoracic echocardiography (TTE) has lower sensitivity (81%) and specificity (61%) in diagnosing PH with underlying lung disease (Group 3) [17]. Indicators like heightened intrathoracic gas, lung tissue consolidation, thoracic cage expansion, and changes in heart position diminish the efficacy of TTE. The most common limitation is that TTE estimates pulmonary artery systolic pressure rather than pulmonary artery pressure, which is used to define PH [18]. Inaccuracies may arise while accurately measuring the peak TR velocity signal. If the Doppler signals are of low quality or imprecise due to improper Doppler alignment caused by eccentric jets, this can lead to both overestimation and underestimation [19].

CT scan, despite being a very good assessment tool, provides limited hemodynamic information. Exposure to ionizing radiation is another drawback of CT scans. Guidelines recommend the use of V/Q scintigraphy to diagnose CTEPH [20]; however, limited spatial resolution and ionizing radiation are some limitations to its use.

## 3 LITERATURE REVIEW

We conducted a literature search in PubMed, Google Scholar, and Cochrane database to identify the literature explaining the role of cardiac MRI in pulmonary hypertension. We have included studies published between 2007 and 2022 (Table **2**) explaining the use of cMRI in PH. We have included 8 full text articles, including 5 prospective studies, 2 retrospective studies and 1 systematic review and meta-analysis.

## 4 RESULTS

A retrospective study conducted by Abe *et al*. involved 84 patients with connective tissue disease. 65 out of 84 participants had RHC confirmed PH in whom the cMRI measured right ventricular end-diastolic dimension index (RVEDDI) was independently linked with higher mortality (hazard ratio 1.24; 95% CI: 1.08–1.46; *P* = 0.003). Thus, by using cMRI to assess the RVEDDI, we can predict mortality rates in patients with PH caused by connective tissue disease. However, further study is necessary to confirm its reliability [21]. In 2022, Alabed *et al*. conducted a meta-analysis including 1938 participants from 22 studies. The findings indicated that cardiac MRI assessment of the right ventricular ejection fraction and right ventricular end-systolic volume index could be utilized as a significant clinical endpoint for PAH therapies. This study offers compelling evidence in favor of using cMRI as a clinically meaningful outcome in studies of PH treatments. cMRI may be a crucial tool for determining clinical deterioration and estimating mortality [22]. A prospective cohort study conducted by Alabed *et al*. in Europe, which involved 723 patients with PH confirmed with RHC, found that the accuracy of predicting 1-year mortality in PAH can be improved with the use of cardiac MRI. The highest risk of mortality is indicated by abnormalities in the end-systolic interventricular septum and end-diastolic left ventricle [23]. According to a study conducted by Alkhanfar and colleagues in 2023 [25], a prospective cohort of 167 patients with complete MRI and RHC data showed that the PAH MRI model for chronic lung disease had a sensitivity of 92.3% and a specificity of 70.2%. According to Alandejani *et al*.'s prospective cohort study in 2022, which included 311 participants, RVEF measured by cMRI is an independent predictor of 1-year mortality in patients with PAH. This study suggests that there are certain measures that evaluate the function of the right ventricle (RV), such as the right ventricular ejection fraction (RVEF), RV end-systolic volume, and the right atrial (RA) area. These measures are useful in predicting outcomes, but only the RV metrics can indicate a low-risk status [25]. A prospective cohort study conducted by Simpson *et al*. in 2022 involved 89 participants. All patients underwent RHC within 24 hours of cMRI. The study found that a 10% increase in the ventricular mass index (VMI) on cardiac MRI resulted in a 54% higher likelihood of a new diagnosis of PAH. Moreover, a VMI greater than 0.37 showed a 90% positive predictive value (PPV) for PAH. By using cMRI to measure VMI and other RV mass metrics, it is possible to predict mild PAH that has been confirmed through right heart catheterization. This prediction is based on the diagnostic threshold established by the Sixth World Symposium on PH [26]. In 2022, Zhang *et al*. performed a retrospective cohort study with 64 participants who underwent both RHC and cMRI within 7 days. The aim of the study was to determine the sensitivity and specificity of diagnosing PAH with a mean pulmonary artery pressure >25mmHg using the right ventricular end-systolic refractory index (RVESRI) >1.35. The results showed a sensitivity of 97.8% and a specificity of 83.3%. Using cMRI, RVESRI provides a straightforward and consistent way to evaluate the hemodynamics and right ventricular function of CTEPH patients [27]. Cerne *et al*. conducted a prospective case-control study in 44 PH patients. The study used RHC to calculate patients' pulmonary vascular resistance (PVR). The velocity of blood flow was measured by 4D flow MRI in the main, left, and right pulmonary arteries. Ejection fraction and end-systolic and diastolic volumes were measured. The scientists assessed these parameters both within and across various pulmonary hypertension (PH) subcategories. The results of the study propose that 4D flow MRI has the potential to identify pulmonary arterial hypertension (PAH) and changes in pulmonary vascular resistance (PVR) [28].

## 5 DISCUSSION

cMRI is a promising adjunctive tool for diagnosing PH. It helps visualize RV structure and function and assess the patient's prognosis by analyzing functional and structural data and velocity information from MRI [29]. MRI is an excellent method for tracking the progress of patients with PAH over time. It is highly reproducible. MRI is sensitive to changes in morphological, functional, and flow-related factors, making it a valuable tool in studying PAH [30]. The structural and functional cardiac status is obtained from cine cardiac MRI, and velocity data is obtained from black-blood and phase contrast MRI [29].

### 5.1 Cine Cardiac MRI

Cine MRI involves capturing a series of cardiac-gated balanced steady-state free precession (bSSFP) images that include both ventricles from base to apex, usually in the short-axis plane. Short-axis images can be used to calculate various volumes and functions of the heart, including RV and LV mass, ejection fraction, end-systolic volume, end-diastolic volume, and stroke volume of both ventricles. Ventricular Mass Index (VMI), which is the ratio of RV and LV mass, can also be calculated. Intraventricular septal angle, the predictor of mortality in patients with PH, can also be measured [29, 31].

### 5.2 RV Function and Diagnosis of PH

Measuring the right ventricle (RV) in pulmonary hypertension (PH) poses challenges due to diverse remodeling and abnormal septal motion. The most reliable and precise method for accurate assessment of ventricular volumes, mass, and identification of motion irregularities is cardiac MRI with cine imaging [4]. Among volumetric parameters, the right ventricular end-diastolic volume (RVEDV) indexed to body surface area is most strongly linked to mortality in PH, with a hazard ratio of 4.20 and a P-value of .016 [6]. It is crucial to meticulously monitor the short-axis stack during diastole to avoid underestimating the RVEDV, especially considering the potential expansion of the enlarged RV beyond the atrioventricular groove, known as the “shoulder” of the RV [32].

It's noteworthy that three-dimensional echocardio-graphy tends to underestimate ventricular volumes, while cardiac CT tends to overestimate them compared to MRI [33]. For assessing overall systolic function, the right ventricular ejection fraction (RVEF) is commonly employed, with a value below 40% indicating RV failure, closely associated with elevated pulmonary vascular resistance [6]. The determination of RV mass through cardiac MRI aligns with mass derived from explanted hearts after transplantation [34]. Additionally, the ventricular mass index (VMI), calculated by dividing the right ventricular (RV) mass by the left ventricular (LV) mass, serves as a valuable biomarker for diagnosing PH [35].

### 5.3 Interventricular Coupling

In individuals with advanced pulmonary hypertension (PH), the right ventricle exhibits a slower contraction rate compared to the left ventricle due to pressure overload. This results in the right ventricle systole extending into the early diastole phase of the left ventricle, causing mechanical asynchrony between the right ventricle's free wall and septum, as well as between the free walls of the right and left ventricles. Due to this inefficient contraction of the right ventricle, the shortening of the right ventricle persists for up to 59 milliseconds after the closure of the pulmonary valve [36]. Interventricular septal bowing is recognized as one of the most significant prognostic factors in PH, particularly when the mean pulmonary arterial pressure exceeds 67 mm Hg [37].

### 5.4 Myocardial Strain and Myocardial Feature Tracking

Myocardial strain refers to the degree of deformation in a myocardial segment from its original length. While cine steady-state free-precession (SSFP) sequences are commonly employed for right ventricle (RV) volume and ejection fraction analysis, quantifying myocardial deformation can identify early abnormalities and carry prognostic implications in pulmonary hypertension (PH), even in cases with preserved ejection fraction [38]. Changes in myocardial strain may manifest despite a preserved ejection fraction, aiding in the early detection of right ventricular decompensation and showing associations with disease severity [39, 40].

Myocardial feature tracking (MFT) is a technique that utilizes a block-matching approach on standard cine SSFP sequences to analyze myocardial motion and infer cardiac deformation. This method is applicable to any cardiac chamber and has demonstrated promising results, showing a strong correlation with cardiac MRI tagging and speckle-tracking echocardiography. Furthermore, the interobserver agreement using MFT has been found to be excellent [4].

### 5.5 Strain Analysis, RV, and PH

While there is limited information regarding the application of myocardial feature tracking (MFT) in the right ventricle (RV), it can contribute to assessing the risk in individuals with pulmonary hypertension (PH) and identifying early RV dysfunction, even in cases where the RV ejection fraction is maintained. Currently, the recommended method for evaluating RV dysfunction is through the use of RV global longitudinal strain. Some researchers have observed significantly lower RV global longitudinal strain, global circumferential strain, and strain rate in PH patients compared to healthy individuals, and these alterations are linked to the severity of the disease. In individuals with preserved ejection fraction and PH, abnormalities in RV global circumferential strain rate are evident, suggesting the potential of MFT in detecting early RV decompensation [38, 41].

### 5.6 Black-blood Imaging

Cardiac gated black blood imaging is a technique that uses a cardiac gated dual inversion recovery fast spin echo sequence to eliminate the signal from flowing blood [42]. Black blood imaging provides high spatial resolution images, which are crucial in evaluating the morphology of heart boundaries and vessel walls. The vessel appears black when blood flow is rapid, a contrast between the vessel's inside and its walls. This method is effective for fast-flowing vessels like the aorta but not so much when blood is turbulent or moving slowly, particularly at vessel division points and in patients with PH [42]. Nonetheless, numerous studies propose the utilization of slow flow occurrences in the pulmonary artery among individuals with pulmonary hypertension (PH). A cohort of 233 patients who were under suspicion of having PH underwent black-blood imaging, revealing encouraging diagnostic outcomes. The examination demonstrated a sensitivity of 86% and specificity of 85%, successfully identifying dilated major pulmonary arteries and regions with reduced blood velocity [5].

### 5.7 Phase contrast MRI

#### 5.7.1 Cardiac Output and Flow Profile

Assessing hemodynamic parameters like cardiac output, flow profile, forward flow, backward flow, average velocity, and peak velocity can be achieved through phase contrast MRI. Various MRI techniques are accessible for measuring cardiac output and stroke volume. Phase contrast imaging provides a reliable method for evaluating blood flow in the pulmonary artery and aorta. Simultaneously, MR volumetry stands as a validated and repeatable approach for gauging alterations in the volume of both the right and left ventricles under both healthy and diseased conditions [43, 44].

#### 5.7.2 Pulmonary Artery Stiffness

Increased vascular resistance in the pulmonary artery as a result of stiff vessels increases RV workload and remodeling. Balanced steady-state free precession (bSSFP) accurately measures pulmonary artery stiffness. Pulmonary artery stiffness can be measured during the cardiac cycle by calculating area change and relative area change (RAC) of the pulmonary artery trunk. A connection between pulmonary arterial stiffness and pulmonary vascular resistance was discovered in a study of 134 individuals with PH. The study found that RAC is linked to negative outcomes (with a p-value of less than 0.05). It's possible that mild PH could affect RAC since compliance and resistance have an inverse-linear connection, with minor increases in pulmonary vascular resistance causing significant reductions in compliance [45]. It has been demonstrated that noninvasively assessed reduced RAC in individuals with PAH predicts mortality [46].

### 5.8 Late Gadolinium Enhancement

Late gadolinium enhancement is used for myocardial tissue characterization, which is a prognostic marker in patients with PH [3]. Gadolinium-based contrast is used to identify areas of the heart that have scar tissue, fibrosis, or have suffered from a heart attack. This contrast is distributed based on the amount of space outside the cells, resulting in a higher concentration of gadolinium and increased signal intensity in these areas. In people with PAH, hyperenhancement is typically present at the RV insertion point, which is associated with poorer clinical status and survival [47, 48]. It is suggested that hyperenhancement has a stronger association with the outcome than fibrosis in the RV insertion alone, particularly if the fibrosis extends into the interventricular septum [48]. T1 mapping: In individuals with PH, T1 mapping enables quantitative evaluation of myocardial tissue alterations, which may be a more sensitive indicator than late gadolinium enhancement [49].

### 5.9 MR Angiography (MRA)

Contrast-enhanced magnetic resonance angiograms provide a comprehensive view of vessel alterations in idiopathic pulmonary arterial hypertension (IPAH) and can highlight thromboembolic material in chronic throm- boembolic pulmonary hypertension (CTEPH). Different angiographic patterns are evident in various pulmonary hypertension (PH) types. IPAH exhibits dilated arteries with distal vessel pruning, while PH associated with chronic obstructive pulmonary disease presents distinctive splayed vessels [50]. Contrast-enhanced magnetic resonance angiography (CE-MRA) stands out as a highly effective diagnostic method for detecting both proximal and distal chronic thromboembolism (CTE), with an overall sensitivity of 98% and specificity of 94%. Although CE-MRA demonstrates lower sensitivity for central vessel disease, combining images with unenhanced MRA enhances sensitivity. Compared to contrast-enhanced pulmonary angiography (CTPA), CE-MRA identifies more stenoses, post-stenosis dilatations, and occlusions. Furthermore, perfusion images from CE-MRA show a 92% sensitivity in diagnosing CTE [51].

### 5.10 Role of cMRI in Determining Prognosis

The increasing recognition of MRI as a dependable, consistent, and responsive biomarker underscores its significance in monitoring patients, evaluating risk, and assessing the efficacy of treatment [52, 53]. In pulmonary hypertension (PH), low cardiac output elevated right atrial pressure, and increased pulmonary vascular resistance serve as acknowledged indicators of an unfavorable prognosis. Studies indicate that cardiac MRI (cMRI) can precisely determine right ventricular (RV) volume, stroke volume, ejection fraction, and left ventricular (LV) volume, offering predictive insights into mortality and treatment outcomes in idiopathic pulmonary arterial hypertension (PAH). Furthermore, research reveals that pulmonary arterial relative area change (RAC), as assessed by cMRI, is a predictive factor for mortality in both PAH and a broader group of patients with PH, highlighting the prognostic significance of pulmonary arterial stiffness [45, 52]. The potential advantages and disadvantages of cMRI in PH are summarized in Table **3**.

### 5.11 Advantages and Disadvantages of cMRI in PH

**Fig. (2):**
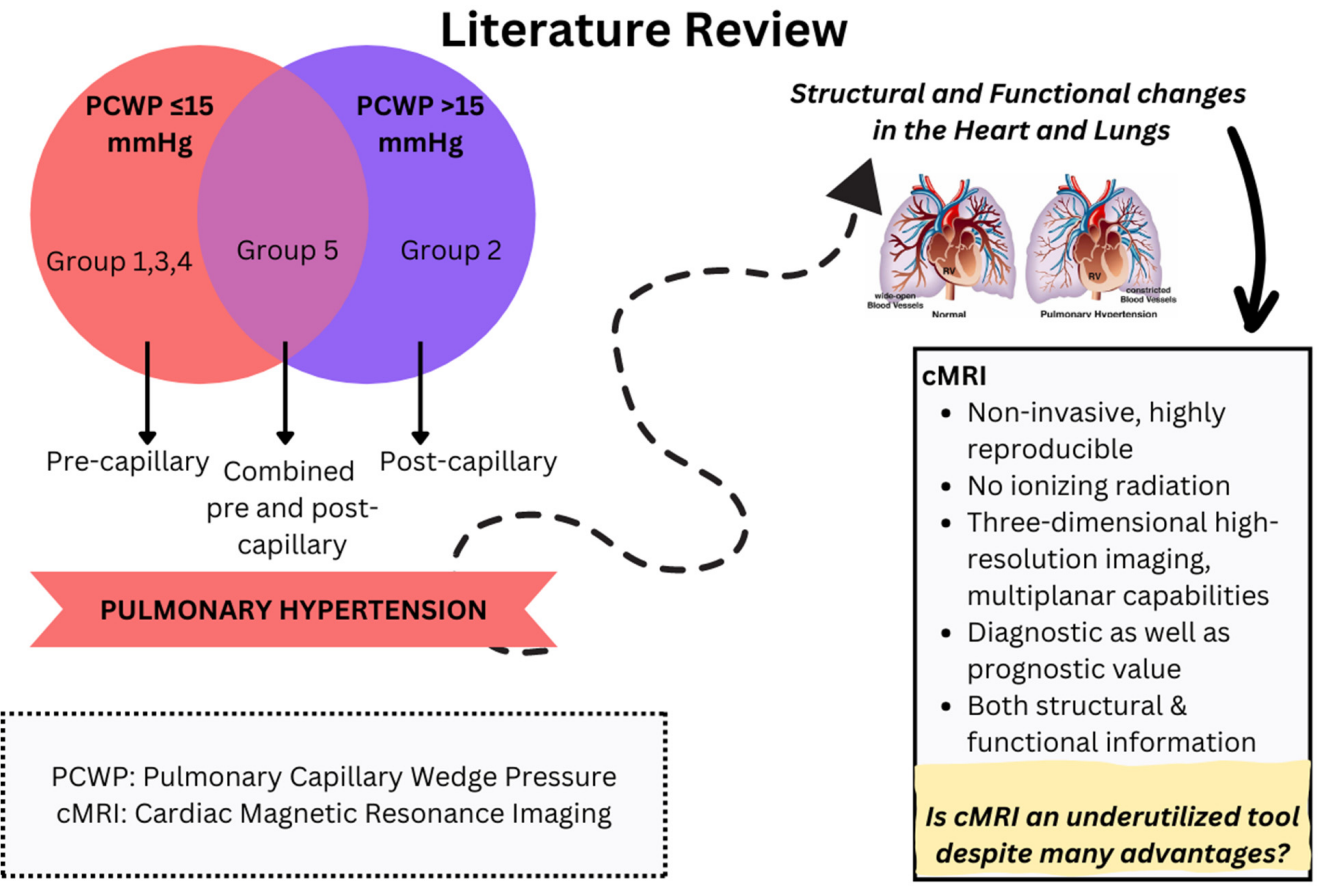
Summarizes the manuscript in a graphical form.

Although there are a few limitations with cMRI, its capability to diagnose, monitor treatment response, and predict mortality makes it an excellent tool to use in patients with PH. When used properly, the advantages significantly outweigh the drawbacks [4,53-55] (Fig. **2**).

## CONCLUSION

Pulmonary arterial hypertension is a serious disease that is best diagnosed currently through invasive hemodynamic measurements. However, noninvasive methods such as cardiac MRI (CMR) can provide useful diagnostic and prognostic information. MRI is particularly useful as it can assess both the pulmonary and cardiac systems, potentially reducing the need for invasive procedures or radiation exposure. MR metrics of RV function are more precise and reproducible than TEE, making it a promising tool for the initial workup and long-term follow-up of PH patients. Thus, we suggest the need for more extensive research to use this noninvasive tool to manage patients with PH.

## Figures and Tables

**Table 1:** Commonly used imaging modalities in pulmonary hypertension and their limitations.

Diagnostic Modality	Limitations
RHC	• Limited assessment of right ventricle condition • High risk of complications
Echocardiography	• Operator dependence • Effect of body habitus • Geometric assumption • Difficulty in quantification • Limited utility for the visualization of pulmonary artery
CT scan	• Limited hemodynamic information • Exposure to ionizing radiation
V/Q scintigraphy	• Limited spatial resolution • Exposure to ionizing radiation

**Table 2:** shows some relevant literature searches regarding cardiac magnetic resonance imaging use in pulmonary hypertension.

Study Population	Study Design and Sample Size	Authors	Inferences
Patients with connective tissue disease who underwent both cMRI and RHC from 2008 to 2018	Retrospective cohort, 84	Abe *et al*. [21]	RVEDDI calculated by cMRI served as a strong predictor of mortality.
Pulmonary hypertension Group 1- 51% Group 2 and 3- 20% Group 4- 26% Group 5- 3%	Systematic review and meta-analysis, 1938(22 studies)	Alabed *et al*. 2021 [22]	Cardiac MRI can be used as the relevant clinical endpoint for therapies of PAH by assessing the right ventricular ejection fraction and right ventricular end-systolic volume index. These parameters are used to predict mortality and serious outcomes in PAH.
Newly diagnosed pulmonary hypertension Group 1- 96% (Idiopathic 27%, congenital heart disease 16%, connective tissue disease 46%) Group 2,3,4,5- 4%	Prospective cohort, 723	Alabed *et al*. 2022 [23]	Prediction of 1-year mortality risk in PAH is more accurate with cardiac MRI.
Chronic lung disease patients suspected of PH -Group 3	Prospective cohort, 167	Alkhanfar *et al*. 2023 [24]	Chronic lung disease- PAH MRI model had a sensitivity of 92.3%, specificity of 70.2%, PPV=77.4%, NPV=89.2%
Pulmonary hypertension	Prospective cohort, 311	Alandejani *et al*. 2022 [25]	Right ventricular ejection fraction (RVEF) measurement *via* cardiac MRI was the independent predictor of 1-year mortality in patients with PAH.
Suspected cases of PH Group 1- 59 (Idiopathic -20, systemic sclerosis- 39)	Prospective cohort, 89	Simpson *et al*. 2022 [26]	For every 10% increase in the ventricular mass index (VMI) on cardiac MRI, the odds of a new diagnosis of PAH increased by 54%, and VMI >0.37 yielded 90% PPV for PAH.
Chronic thromboembolic pulmonary hypertension (Group -4)	Retrospective cohort, 64	Zhang *et al*. 2022 [27]	Sensitivity and specificity for right ventricular end-systolic refractory index (RVESRI) >1.35 to diagnose PAH with mean pulmonary artery pressure > 25mmhg is 97.8% and 83.3%
44 PH patients (56% female); 25 controls (36% female)	Prospective case-control	Cerne *et al* [28]	4D flow MRI can detect PAH and monitor PVR changes. In people with PAH, the blood flow velocity is significantly lower in the left and right pulmonary arteries compared to those without the condition.

**Table 3:** Advantages and potential disadvantages of utilizing cardiac magnetic resonance imaging in pulmonary hypertension.

Advantages [4]	Disadvantages [54, 55]
• Multiplanar capabilities, lack of geometric assumptions, high contrast-to-noise ratio, and function • Unparalleled resolution • Three-dimensional imaging capacity • Noninvasive • No ionizing radiation • Ability to depict soft tissues due to High contrast resolution • Good assessment of post-therapy cardiac remodeling • High temporal and spatial resolution • It can be used for treatment selection	• Prone to artifacts • Limited availability and clinical experience • Long scan times and patient discomfort • The radio frequency field, which is used for excitation, can induce heating of tissue and implanted devices. • Gadolinium-containing contrast agents are risk factors for the development of nephrogenic fibrosing dermopathy in patients with stage 3 or higher chronic kidney disease and should be avoided in those with advanced renal failure. • Patients with pulmonary hypertension may find it difficult to complete the test because of the time and breath-holding required. Claustrophobia is a serious problem that can be overcome in the majority of patients by using mild sedation, although this is often inappropriate in pulmonary hypertension patients.

## LIST OF ABBREVIATIONS

PH: Pulmonary Hypertension
PA: Pulmonary Artery
cMRI: Cardiac Magnetic Resonance Imaging
LV: Left Ventricle
RV: Right Ventricle
